# Draft genome sequence of five strains of family Lactobacillaceae isolated from a seasoning liquid of Hiroshimana old pickle

**DOI:** 10.1128/mra.00521-25

**Published:** 2025-09-04

**Authors:** Toshikazu Suenaga, Erina Shimura, Jean De Dieu Shema, Takehiko Gotoh, Wataru Nishijima, Satoshi Nakai

**Affiliations:** 1 Graduate School of Advanced Science and Engineering, Hiroshima University, Higashi-Hiroshima, Hiroshima, Japan; 2 Environmental Research and Management Center, Hiroshima University, Higashi-Hiroshima, Hiroshima, Japan; Portland State University, Portland, Oregon, USA

**Keywords:** Lactobacillaceae, *Lactiplantibacillus plantarum*, *Pediococcus ethanolidurans*, *Pediococcus parvulus*, *Companilactobacillus alimentarius*, draft genome, Hiroshimana old pickle

## Abstract

We report the draft genome sequences of five strains isolated from a seasoning liquid of *Hiroshimana* old pickle. The strains were identified as *Lactiplantibacillus plantarum, Pediococcus ethanolidurans, Pediococcus parvulus,* and *Companilactobacillus alimentarius*. These strains may contribute to the taste of traditional Japanese pickle and have the potential to provide probiotic benefits.

## ANNOUNCEMENT

Lactic acid-producing bacteria are one of the predominant species of fermented pickles, contributing to the long-term preservation and unique flavor (1). *Hiroshimana* (*Brassica rapa var. toona subvar. hiroshimana*) pickle is a traditional fermented food of Hiroshima prefecture in Japan and is pickled in 2% to 5% brine. We previously demonstrated that *Aurantiochytrium* sp. strain L3W, which produces polyunsaturated fatty acids (PUFAs) (2), could grow on food wastes, such as *Hiroshimana* old pickle, to produce an environmentally friendly feed (eco-feed) usable as a source of PUFAs for livestock industry (3). During the cultivation of *Aurantiochytrium* sp. at pH 4, the microorganisms of family Lactobacillaceae survived and existed in an eco-feed (2). There are several papers reporting the probiotic effect of *Lactobacillus*-related species on livestock (4, 5). We successfully isolated five strains of family *Lactobacillaceae* from the *Hiroshimana* old pickle seasoning liquid (3) and report on the genomes here to investigate the metabolic capabilities.

The *Hiroshimana* old pickle seasoning liquid was diluted stepwise and spread onto MRS agar plates (Fujifilm, Tokyo-Japan) containing 10 g/L-CaCO_3_. The plates were then incubated at 25°C until colonies formed. The grown colonies were isolated and aerobically incubated with MRS liquid medium (3). For DNA extraction, cultured cells, which had been incubated at 25°C for 3 days, were collected by centrifugation and washed twice with phosphate-buffered saline. Cells were lysed with pH 8-TE buffer containing 0.33%-SDS, and nucleic acids were extracted and purified by a phenol-chloroform method (6). The contained RNA was degraded with RNase A (Takara Bio, Shiga-Japan), and the DNA was precipitated by ethanol. The DNA was enzymatically sheared into short fragments. The library was prepared from the obtained fragments using the Rapid Plus DNA Lib Prep Kit for Illumina v2 (ABclonal, Hubei-China), MGIEasy Circularization Kit, and MGIEasy Universal Library Conversion Kit (MGI_Tech. Shenzhen-China) for end repair, A-tailing, adapter ligation, and circularization. The library was sequenced with 150 × 150 paired-end sequencing mode on DNBSEQ T7 platform, provided by the sequencing service (Novogene, Beijing-China). Poor-quality reads (<Q20) and sequencing adapters were removed with fastp_v.0.23.2 (7) and assembled with MEGAHIT_v.2.1.9 (8). The genome sequence was annotated using the DDBJ Fast Annotation and Submission Tool_v.1.6.0 (9), and the rRNA-encoding genes were further identified by barrnap_v.0.9 (10). Completeness of the genome was identified using BUSCO_v.1 (11). All tools were run with default parameters unless otherwise specified. The information of sequencer outputs and genomes is summarized in Table 1.

**TABLE 1:** Summarized information of draft genomes of five strains

Strain name	SHA2	SHB4	SHB5	SHB9	SHB14
Species name	*Lactiplantibacillus plantarum*	*Pediococcus ethanolidurans*	*Pediococcus parvulus*	*Companilactobacillus alimentarius*	*Pediococcus ethanolidurans*
Percent identity of BLAST search (%)	99.94	100	100	99.87	100
Genome size (Mbp)	3.41	2.17	2.20	2.56	2.18
GC content (%)	44.2	37.4	38.6	35.3	37.4
Number of contigs	153	145	212	100	166
N50 of assembly (kbp)	91.7	56.0	82.2	76.7	56.1
Number of rRNA	7	6	5	7	5
Number of tRNA	66	62	61	55	63
Num. of predicted genes	3,248	2,134	2,111	2,542	2,124
Output number of reads (×10^6^ reads)	7.98	8.37	11.0	8.79	9.92
Output bases (Gbp)	1.20	1.26	1.65	1.32	1.49
Coverage (×)	351	580	750	516	683
BUSCO score [%]	97.5	95.0	95.0	97.5	95.0
Acc. num. of genome assembly	BAAHNJ000000000	BAAHNF010000000	BAAHNG010000000	BAAHNH010000000	BAAHNI010000000
Acc. num. of BioSample	SAMD00912541	SAMD00912542	SAMD00912543	SAMD00912544	SAMD00912545
Acc. num. of SRA	DRR681810	DRR681811	DRR681812	DRR681813	DRR681814

**Fig 1:**
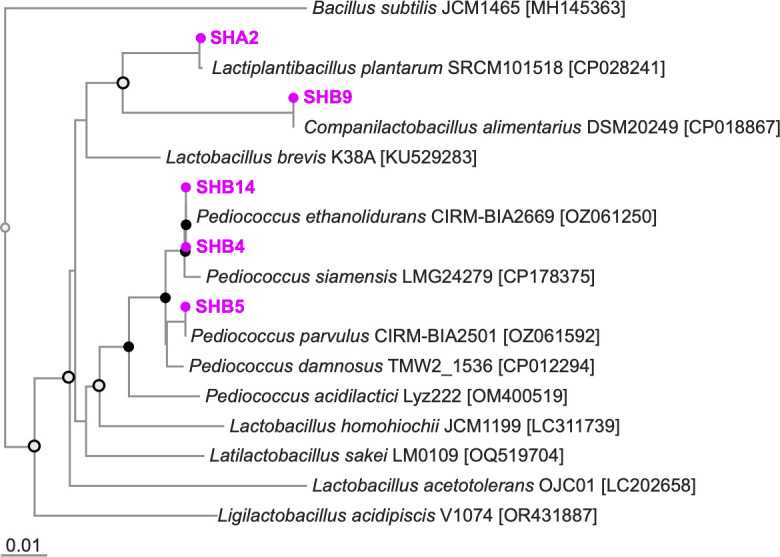
Phylogenetic tree based on the full-length 16S rRNA gene. The strains analyzed in this study are shown in bold. Bootstrap values are derived from 100 replicates, indicated as >75 (closed circles) and in the range of 50–75 (open circles) at each node. The phylogenetic tree was generated using Archaeopteryx.js_v.2.0.0 and then modified visually using Affinity Designer 2_v.2.6.2.

The draft genomes were assembled with over 350× coverage, resulting in 100 to 212 contigs. The phylogenetic relationships of the isolated strains based on the full-length 16S rRNA were calculated with MAFFT_v.7 (12) and are shown as Fig. 1. Nucleotide BLAST search (13) of the 16S rRNA gene revealed that two strains (SHB4 and SHB14) were identified as *Pediococcus ethanolidurans*, while strains SHA2, SHB5, and SHB9 were identified as *Lactiplantibacillus plantarum*, *Pediococcus parvulus*, and *Companilactobacillus alimentarius*, respectively. The percent identity of the BLAST search ranged from 100% to 99.87%. The genomic information revealed in this study will help to elucidate probiotic effects of eco-feeds for livestock in the future.

## Data Availability

The sequencing data have been deposited at DDBJ as the BioProject (PRJDB20731) and BioSample (SAMD00912541–SAMD00912545). The accession numbers of raw reads, genome, and assembly for each strain are listed in Table 1.
